# A Rare Abdominal Pain Associated With Chronic Intestinal Schistosomiasis Diagnosed Endoscopically: A Case Study From Saudi Arabia

**DOI:** 10.7759/cureus.58614

**Published:** 2024-04-19

**Authors:** Saeed Nasser Alsharif, Ali Saleh Alshamrani, Dawlah Hadi Asiri, Salihah Yahya Al-Mani

**Affiliations:** 1 Gastroenterology, Armed Forces Hospital - Southern Region (AFHSR), Khamis Mushait, SAU; 2 Internal Medicine, Armed Forces Hospital - Southern Region (AFHSR), Khamis Mushait, SAU

**Keywords:** clinical case report, saudi arabia, histopathology, colonoscopy, chronic intestinal schistosomiasis

## Abstract

Chronic intestinal schistosomiasis (CIS) refers to the long-term effects of infection with Schistosoma parasites in the intestines. This condition typically develops after repeated or prolonged exposure to contaminated freshwater containing Schistosoma eggs. The current study reports a case of an adult male, who complained of abnormal abdominal and anal pain for a month and had a medical history of complex perianal fistulae. The endoscopic investigation revealed different degrees of hyperemia, concentrated in the sigmoid colon and rectum. Lesions were localized in the rectum and sigmoid colon. Yellow granular hyperplasia, whether concentrated or dispersed, single or multiple polyps, along with observations of mucosal congestion, edema, faint vascular striations, erosions, superficial ulcers, and scattered petechial hemorrhages were noted. Also, the segmented areas of the colon had different degrees of inflammation. The microscopic histopathological analysis showed a culprit of surgical scar tissue. The granulomas harbored Schistosome parasites at the submucosal depth. Also, an erosion in the colonic mucosal tissues accompanied by lymphoplasmacytic and micro-abscess infiltrates was seen. A Schistosoma bilharzial ova was observed in the granuloma at the submucosal level. Endoscopic and histopathological investigations are useful tools to differentiate between CIS and Crohn's disease. These tools can distinguish CIS from Crohn's disease. Early detection and treatment are essential to prevent the progression of the disease and minimize long-term complications.

## Introduction

Schistosomiasis, or bilharzia, is a trematode parasitic illness caused by numerous types of flatworms known as Schistosomes. These parasites infect people mostly by contact with polluted water containing the parasite's larvae (*cercariae*) [1]. Chronic intestinal schistosomiasis (CIS) is a public health issue associated with persisting in tropical and subtropical climates caused by parasitic flatworms of the Schistosoma genus [2]. When exposed to polluted waters, the larvae of these parasites penetrate human skin, move through the circulation, and finally lodge in the intestines' mesenteric veins. Adult worms lay eggs, which can cause a variety of symptoms such as stomach discomfort, diarrhea, and bloody stool [3]. An acute infection involves a swimmer's itch and acute schistosomiasis syndrome. CIS may be asymptomatic or present with abdominal pain, diarrhea, and bloody stool. Weight loss, iron-deficiency anemia, eosinophilia, hypoalbuminemia, constipation, and rectal prolapse may occur [4]. Ova deposits in the colonic submucosa, smooth muscle, and sub-serosa cause an immunological response and local chronic inflammation with or without fibrosis. In some patients, secondary ischemia may result in polyps, ulceration, mucosal bleeding, and even strictures. These changes make it difficult to distinguish between intestinal schistosomiasis and inflammatory bowel disease (IBD) or Crohn's disease (CD) [4]. The main species of Schistosoma causing CIS in Saudi Arabia is *Schistosoma mansoni* [5]. In Saudi Arabia, the estimated prevalence dropped to 0.025% in 2010, compared to 6.5% in 1982 [6]. Another seven-year retrospective study, from 2014 to 2020, reported a cumulative incidence rate of 2.155/100000 CIS cases among the Saudi population, where the highest rate was in 2015, mostly among males [5]. Both clinical forms (urinary and CIS) exist in Saudi Arabia in seven out of 13 regions, where Mecca has a relatively high number of cases [5]. Overall, while CIS remains a concern in certain regions of Saudi Arabia, concerted efforts in prevention, control, and research are essential for reducing the burden of this parasitic disease on public health in the country. In the current research, we reported rare abdominal pain and weight loss associated with CIS in the Aseer region of Saudi Arabia.

## Case presentation

A 31-year-old Saudi male, medically free, visited the emergency department of the Armed Forces Hospital, Southern Region, Khamis Mushait City, Saudi Arabia, on October 15, 2023. The patient complained of continuous abdominal pain for four weeks. The pain started acutely, localized to the left side of the abdomen, and was intermittent, not radiating or relieved by defecation. It was associated with diarrhea, fresh bleeding per rectum, and anal pain. Diarrhea was a loose stool with four to six bowel motions per day, which did not improve with fasting or urgency; however, it did not cause any sleeping issues. These common symptoms of CIS include diarrhea, abdominal pain, dyspepsia, bloody stool, and malnutrition; however, they are non-specific and might result in misdiagnosis [2]. Symptoms have progressively exacerbated in the last four days. He had neither a fever, a sweet night, nor weight loss. The patient denies any symptoms of melena, hematemesis, nausea/vomiting, loss of appetite, heartburn, dysphagia, skin changes/rash, weight change, jaundice, dark urine, early satiation, abdominal swelling, or fever. Also, no joint pain, palpitation, headache, or dizziness were reported. The patient has been an active smoker for the last 10 years. He had a past medical history of complex perianal fistulae. He denied the use of antibiotics, proton pump inhibitors (PPI), non-steroidal anti-inflammatory drugs (NSAIDs), alcohol and illicit drug abuse, ex-maternal affairs, a family history of IBD, or celiac disease. There was no recent travel history outside his area. The patient mentioned a remote history of swimming in a rainwater pond.

In the initial investigation, the patient looked well, conscious, alert, and oriented, with no pale or jaundice. There was no conjunctival injection or telangiectasia. The external investigation did not detect any abdominal scars, while soft and mild non-localized tenderness was confined to the left side with no organomegaly. Perianal fistulas were detected bilaterally on perianal examination, with a small amount of hematochezia. Also, there was no skin rash or joint swelling.

For the clinical diagnosis of CIS, further laboratory tests were made to investigate any abnormal complete blood count (CBC), liver functions, or basic metabolic panel (Bmp). Furthermore, the antibody reactivity for Schistosome (serum) and *Clostridium difficile* bacterial toxin was performed as well. The results showed normal blood analysis, except for a slight increase in gamma-glutamyl transpeptidase (GGT) (66 IU/L) and mild eosinophilia. Table 1 summarizes the laboratory results.

**Table 1:** Full blood analysis of the studied case Hb, hemoglobin; HCT, hematocrit; Plt, platelets count; ESR, erythrocyte sedimentation rate; CRP, C-reactive protein; Tb, total bilirubin; Db, direct bilirubin; ALT, alanine transaminase; AST, aspartate transaminase; ALP, alkaline phosphatase; GGT, gamma-glutamyl transpeptidase; Pt, prothrombin time; aPTT, activated partial thromboplastin time; INR, international normalized ratio; Bmp, basic metabolic panel; Na, sodium; K, potassium

Variables	Results	Normal values
Hematology		
WBCs (×10^9^/L)	6.75	4-11
Hb (g/dL)	16.6	14-18
HCT [%]	52.6	38-52
Plt (×10^9^/L)	285	150-400
Neutrophils absolute count (×10^9^/L)	1.79	2-7.5
Lymphocytes absolute count (×10^9^/L)	3.63	1.5-4
Eosinophiles absolute count (×10^9^/L)	0.81	0.1-0.5
Monocytes absolute count (×10^9^/L)	0.31	0.2-1
ESR (mm/hr)	13	<15.5
Liver function		
CRP (mg/L)	11.5	<10
Total protein	Negative	Negative
Tb (µmol/L)	10.5	5.1-20.5
Db (mg/dL)	0.5	1.7-8.6
ALT (U/L)	17	7-55
AST (U/L)	16	8-48
ALP (U/L)	76	32-91
GGT (IU/L)	66	11-55
Pt (seconds)	11.5	11-13
aPTT (seconds)	30	21-35
INR	1	<1.1
Bmp		
Na (mEq/L)	141	136-144
K (mmol/L)	4	3.6-5.1
Microbiology		
Schistosome (serum)	Negative	<1:80
*Clostridium difficile* toxin	Negative	Negative
Ova and parasite	Negative	Negative

The initial diagnosis considered included inflammatory Crohn's disease with complex perianal fistula and various infectious causes such as* C. difficile, Salmonella, Shigella, Escherichia coli *O157:H7, cytomegalovirus (CMV), or herpes simplex virus (HSV) infection.

**Figure 1:**
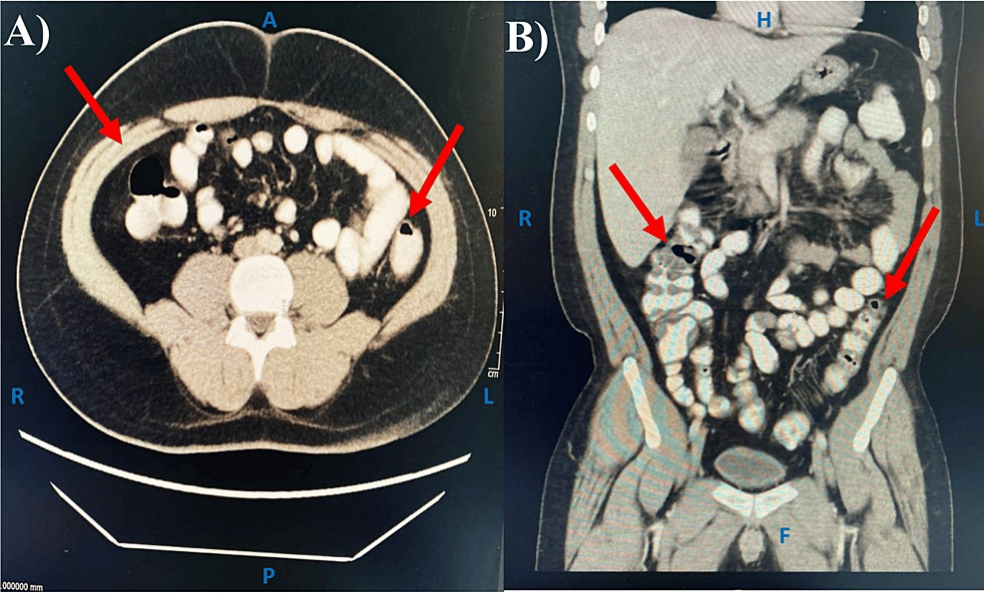
CT scan of abdomen and pelvis with IV contrast Several peri-rectal staining areas are seen in the rectum and the recto-sigmoid region of the colon (red arrows), which indicates inflammation. A) axial view; B) coronal view. A, anterior; P, posterior; R, right; L: left

Further diagnostic imaging via computed tomography (CT) and magnetic resonance imaging (MRI) aimed to pinpoint an accurate diagnosis by investigating the abdominal inflammation areas. The CT scan of the abdomen and pelvis with IV contrast showed an enlarged liver and bulky spleen without any focal lesions. The pancreas size was average and had a tiny hyperdense shadow (0.3 cm). A small precaval lymph node (0.9×0.8 cm) was seen. The colon had patchy areas of relative wall thickening, whereas the rectum had significant soft tissue thickening that extended into the recto-sigmoid region with peri-rectal staining. Finally, a few enlarged local lymph nodes of almost 0.7 cm were noted, which might be an indication of inflammation (Figure 1).

**Figure 2:**
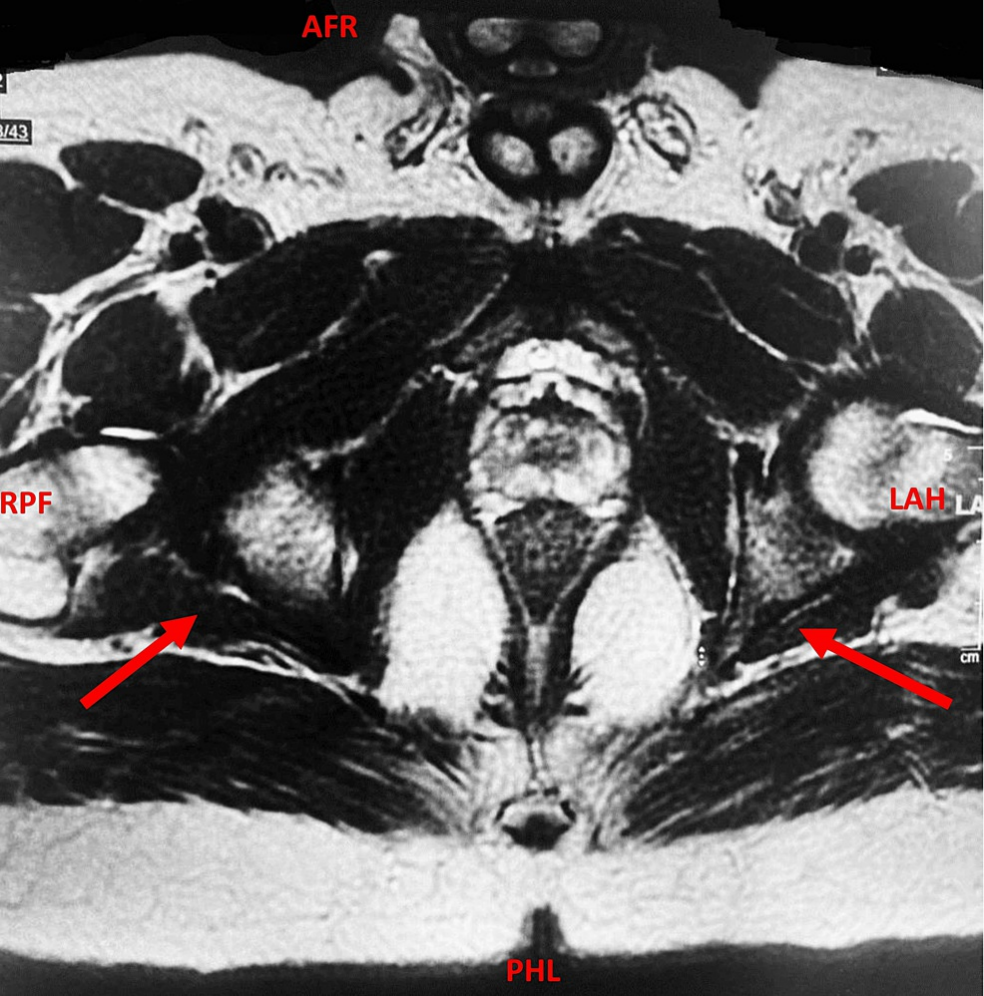
MRI scan of pelvis with IV contrast using a fistula protocol The red arrows indicate an abnormal bilateral perianal inter-sphincteric fistula with internal openings. MRI, magnetic resonance imaging; AFR, anterior-foot-right; PHL, posterior-head-left; RPF, right-posterior-foot; LAH, left-anterior-head

Meanwhile, the MRI of the pelvis with IV contrast using a fistula protocol identified a bilateral perianal inter-sphincteric fistula with internal openings at 5 o'clock and 7 o'clock, along with mild wall thickening in the rectum and sigmoid colon. Fluid collection, trans-sphincteric or supra levator extension, or pelvic lymphadenopathy were not evidenced (Figure 2).

**Figure 3:**
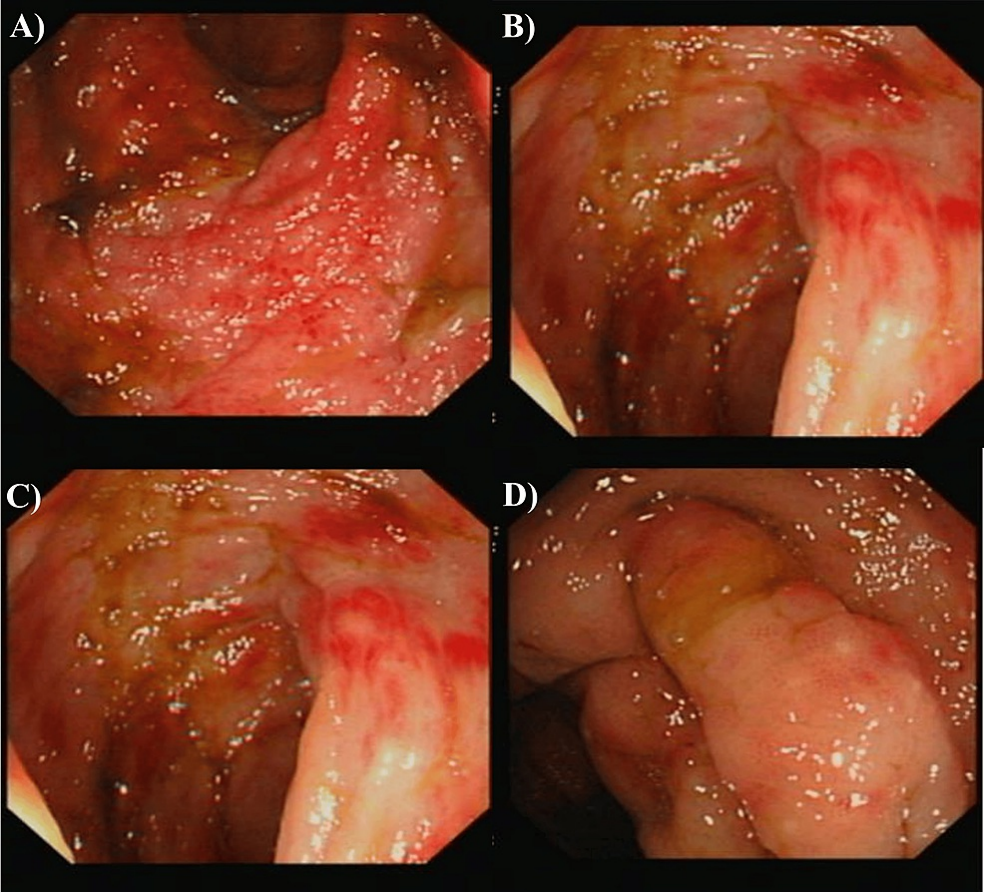
An endoscopic investigation of the diffused hyperemia in the colon and rectum A) Aphthous ulceration, B) skip lesions, C) inflammation started from rectum to cecum, and D) normal terminal ileum

Subsequent colonoscopies comprehensively evaluated the entire colon, including the descending, transverse, sigmoid colon, and rectum. That endoscopy aimed to investigate any lesions, ulcers, or hemorrhage through the digestive tract and to isolate a biopsy for further histopathological diagnosis of Schistosoma ova or parasites. Endoscopic findings unveiled varying degrees of hyperemia, ranging from mild to severe, primarily concentrated in the sigmoid colon and rectum. Lesions predominantly localized in the rectum and sigmoid colon were characterized by features indicative of CIS, such as dense or scattered yellow granular hyperplasia, single or multiple polyps, mucosal congestion, edema, faint vascular striations, erosions, superficial ulcers, and scattered petechial hemorrhages. Additionally, acute and chronic inflammations were noted in segments of the right and left colon, as well as some segmented areas of the colon (Figure 3).

**Figure 4:**
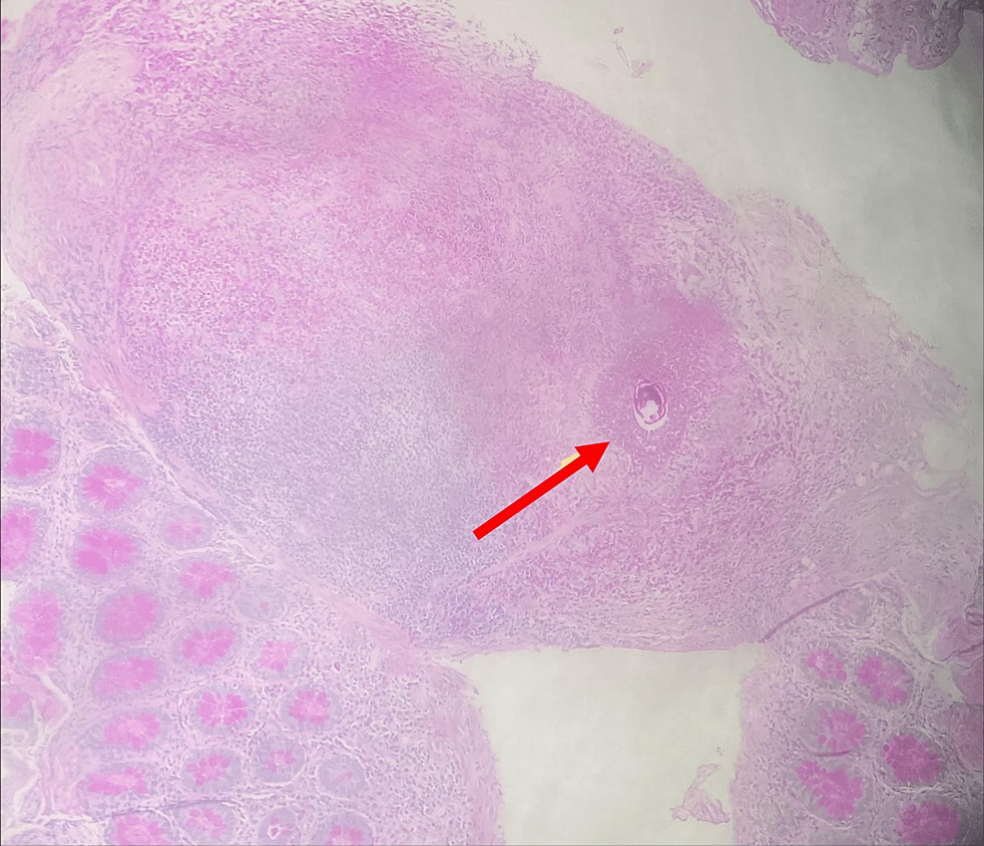
Histopathological analysis of the colon biopsy of the studied case A colon punch biopsy sized 6 mm was taken from the segmented colon. The slide was stained with H&E stains and visualized by Leica DM 2000 microscopy (Leica Microsystems - Danaher Corp., WA, United States). The red arrow shows a clear culprit of surgical scar with an oval-shaped structure consistent with Schistosoma bilharzial ova. H&E, Hematoxylin and Eosin

For histopathological investigation, a biopsy of almost 6 mm was taken from the entire colon at the site of the diffused hyperemia. As shown in Figure 4, a clear culprit of surgical scar tissue was observed. The examined section revealed multiple colonic mucosal tissues that showed erosion with relatively preserved architecture and dense chronic lymphoplasmacytic infiltrates in the lamina propria. Abundant eosinophils with micro-abscess formation and infiltration into the crypts are seen. The focus of granuloma is noted to have epithelioid histiocytes with abundant eosinophils and a rare refractile oval-shaped structure consistent with Schistosoma bilharzial ova. The granulomas contained Schistosome parasites at the submucosal level. The histopathological investigation did not morphologically indicate a specific Schistosoma species. That was diagnosed as a random colonic biopsy showing granulomatous colitis with abundant eosinophils and a rare refractile oval structure consistent with Schistosoma.

The treatment protocol included a regular prescription medication of Praziquantel for two days. The patient's abdominal pain and other symptoms disappeared dramatically with gaining regular weight and good nutrition, as observed in the follow-up visits in the gastroenterology clinics.

## Discussion

Schistosomiasis is a serious public health concern in many parts of the world, particularly in tropical and subtropical countries with little sanitation and clean water. Schistosomiasis prevention tactics include improving access to clean water and sanitation as well as implementing preventative measures including snail management and antiparasitic medication delivery. CIS is a prevalent parasitic illness in tropical and subtropical countries, especially in places with inadequate sanitation and limited access to clean water [7]. However, occasional instances of CIS have been observed in non-endemic locations as a result of travel or migration from endemic areas. These instances frequently present diagnostic hurdles to professionals inexperienced with the condition, resulting in delayed diagnosis and potentially serious sequelae. The diagnosis is based on clinical suspicion, serological testing, stool analysis for parasite eggs, and imaging procedures like ultrasound or CT [8]. However, early identification and treatment are critical for avoiding chronic consequences such as intestinal fibrosis, obstruction, and hepatosplenic disease. Increased awareness among healthcare practitioners and travelers about the risk of meeting uncommon intestinal schistosomiasis patients outside of endemic areas is critical for prompt identification and treatment of this potentially crippling infection.

In the current case report, an adult Saudi male was diagnosed by an endoscopic and histopathological investigation with CIS. The patient suffered from acute abdominal pain, diarrhea, bleeding from the rectum, and anal pain. The endoscopic investigation revealed remarkable hyperemia with lesions in the rectum and sigmoid colon. Furthermore, the histopathological analysis defined some schistosome parasites in the granulomas contained in the submucosa, as indicated by the culprit of surgical scar tissue.

Previous research has yielded similar observations regarding CIS and its differentiation from other inflammatory gastrointestinal disorders. In a retrospective study conducted at Wuhan University, China, 80 cases diagnosed with CIS or CD were examined, with all patients presenting symptoms of abdominal pain and anal hemorrhage [4]. That study showed that anemia, elevated levels of C-reactive protein (CRP), and erythrocyte sedimentation rate (ESR) were more consistently observed among CD patients compared to those with CIS. The distinguishing features between the two conditions included segmental inflammation, aphthous or longitudinal ulceration, and a cobblestone pattern, which were indicative of CD but not CIS. Although Schistosoma ova were not detected in the intestinal tissue upon microscopic histopathological examination, they were present in small and superficial mucosal biopsies. Additionally, various degrees of fibrosis in the submucosa, granulomas, and multinucleate giant cells were common findings in CIS cases.

Another study investigated the endoscopic findings and clinicopathologic characteristics of 46 patients with intestinal schistosomiasis, who were diagnosed with acute, chronic, or chronic active schistosomal colitis [9]. This study observed intact Schistosoma ova in the colonic mucosa of patients with acute schistosomal colitis. Furthermore, various types of polyps including signal polyps, hyperplastic polyps, canalicular adenomas with low-grade intraepithelial neoplastic changes, and tubulovillous adenomas with high-grade intraepithelial neoplastic changes were identified.

Another study from Ethiopia reported the case of a 12-year-old male who was diagnosed with ulcerative colitis ascribed to schistosomiasis and amoebiasis coinfection, which resulted from previous swimming in an infected pond [10]. That was in contrast to the study conducted by Ak et al. (2022), who presented a case of misdiagnosed schistosomal colitis in a 26-year-old Guinean male patient previously diagnosed with ulcerative colitis [11].

The endoscopy and histopathological analysis were useful for the diagnosis of CIS in a 21-year-old Moroccan male; however, the physical examination and laboratory analysis were normal [12]. In that case study, the colonoscopy showed some cecal polypoid lesions, and the histopathological scanning showed obvious schistosomal ova with viable embryos inside [12]. In another case report of an adult Zimbabwe student, the colonoscopy showed numerous lymphoid nodules and intestinal bleeding, and the histopathological examination showed a Schistosoma ova structure in the intestinal crypts [13]. These case studies, in accordance with our findings, highlight the importance of both endoscopy and histopathological examinations in the early diagnosis of CIS to avoid the misdiagnosis of other diseases with similar symptoms.

Despite a significant decrease in schistosomiasis in Saudi Arabia in recent years, it is still recorded in many areas. A previous retrospective study showed that both urinary and intestinal schistosomiasis were present in Saudi Arabia, mostly among males, with the Mecca and Najran regions having the highest infection spots [5]. In a case study from Bisha province, Saudi Arabia, acute intestinal schistosomiasis was diagnosed in nine school-aged children, of whom four had a previous family history of CIS [14].

## Conclusions

In summary, we report an unusual presentation of CIS. Despite similar symptoms with Crohn's disease, endoscopic and histopathological investigations were useful tools to correct its misdiagnosis. Early detection and treatment are essential to prevent the progression of the disease and minimize long-term complications. Many awareness campaigns should be conducted to warn people of the dangers of swimming in polluted water. Also, medical teams in emergency departments must be alerted to the possibility of schistosomiasis infection, which will consequently reduce complications and increase the chances of effective treatment. Raising awareness about CIS in non-endemic areas among clinicians involves regular educational workshops, seminars, and online training about the epidemiology, prevalence, symptoms, clinical presentation, diagnosis, risk factors, and treatment of CIS. Also, clinical evidence-based guidelines and patient education materials might address the diagnosis and management of CIS in endemic and non-endemic areas. By implementing these strategies, clinicians can become more aware of CIS, leading to earlier recognition, diagnosis, and management of the condition.

## References

[1] Di Bella S, Riccardi N, Giacobbe DR, Luzzati R (2018). History of schistosomiasis (bilharziasis) in humans: from Egyptian medical papyri to molecular biology on mummies. Pathog Glob Health.

[2] Shuja A, Guan J, Harris C, Alkhasawneh A, Malespin M, De Melo S (2018). Intestinal schistosomiasis: a rare cause of abdominal pain and weight loss. Cureus.

[3] Colley DG, Bustinduy AL, Secor WE, King CH (2014). Human schistosomiasis. Lancet.

[4] Cai L, Chen Y, Xiao SY (2021). Clinicopathologic features of chronic intestinal schistosomiasis and its distinction from Crohn disease. Am J Surg Pathol.

[5] Zrieq R, Alzain MA, Ali RM (2023). Epidemiological profile of urinary and intestinal schistosomiasis in the Kingdom of Saudi Arabia: a seven-year retrospective study. Trop Med Infect Dis.

[6] Butrous G (2014). Saudi guidelines on the diagnosis and treatment of pulmonary hypertension: schistosomiasis and pulmonary arterial hypertension. Ann Thorac Med.

[7] Aula OP, McManus DP, Jones MK, Gordon CA (2021). Schistosomiasis with a focus on Africa. Trop Med Infect Dis.

[8] Coltart C, Whitty CJ (2015). Schistosomiasis in non-endemic countries. Clin Med (Lond).

[9] Cao J, Liu WJ, Xu XY, Zou XP (2010). Endoscopic findings and clinicopathologic characteristics of colonic schistosomiasis: a report of 46 cases. World J Gastroenterol.

[10] Ketema W, Taye K, Tagesse N (2022). Fulminant hepatitis and ulcerative colitis: case report of Ethiopian child with schistosomiasis and amebiasis co-infection. Int Med Case Rep J.

[11] Ak Ç, Sayar S, Kılıç ET (2022). A Schistosoma colitis case misdiagnosed as ulcerative colitis in a non-endemic area: a case report. Iran J Parasitol.

[12] Koulali H, Zazour A, Khannoussi W, Kharrasse G, Ismaili Z (2022). Colonic schistosomiasis: a case report. World J Gastrointest Endosc.

[13] Gałązka JK, Starownik D, Kasztelan-Szczerbińska B, Cichoż-Lach H (2022). Imported parasitosis as a diagnostic challenge in primary healthcare clinic in the non-endemic region — a case study of schistosomiasis in English division student. Med Res J.

[14] Alqahtani DO, Abbas M, Alshahrani AM, Ibrahim ME (2017). Acute intestinal schistosomiasis among school-aged children presented to King Abdullah Hospital, Bisha province Saudi Arabia: a case series. Trop Biomed.

